# Spastic co-contraction: A misdirected central command

**DOI:** 10.1016/j.cnp.2026.08.005

**Published:** 2026-08-15

**Authors:** Jean-Michel Gracies, Robert J. Kahoud, Maria Vinti, Simon C. Gandevia, David Burke, Maud Pradines

**Affiliations:** a Neuroscience Research Australia and University of New South Wales, Randwick, NSW, Australia; b University of Corsica, Neurorehabilitation Department, Bastia Hospital, France; c UR 7377 BIOTN, Université Paris-Est Créteil (UPEC), F-94000 Créteil, France; d Pediatrics Department, Mayo Clinic Rochester, United states; e Univ. Limoges, HAVAE, UR 20217, F-87000 Limoges, France; f Department of Neurology, Royal Prince Alfred Hospital and University of Sydney, Sydney, Australia

**Keywords:** Spastic co-contraction, Spasticity, Hemiparesis, Isometric contraction

## Abstract

**Objective:**

Antagonist co-contraction triggered by voluntary agonist command in spastic paresis is poorly understood. Using an isometric ankle paradigm designed to minimize movement-related stretch reflexes, we aimed to characterize the severity, modulation, and neurophysiological origin of spastic co-contraction.

**Methods:**

Thirteen subjects with chronic hemi/paraparesis (*n* = 14 limbs) were seated with the ankle at 90° dorsiflexion, and performed submaximal then maximal isometric voluntary ankle plantar- and dorsiflexion efforts, with the knee flexed then extended (gastrocnemii slack then stretched). Surface and intramuscular EMG recordings (the latter using hook wire electrodes) from agonist and antagonist muscles were combined with ankle torque measurements. Co-contraction severity was classified as *absent*, no antagonist EMG activity during agonist effort; *mild*, presence of antagonist EMG activity without torque reversal; or *severe*, antagonist EMG activity associated with torque in the unintended direction.

**Results:**

Antagonist co-contraction occurred in all limbs during dorsi- and plantar flexor efforts. Its severity increased from submaximal to maximal efforts, whether in knee flexion (*p* = 0.046, Wilcoxon) or knee extension (*p* = 0.014). Severe plantar flexor co-contraction was more prevalent with the knee extended than flexed (64% *vs* 0%; *p* = 0.004, McNemar). Antagonist activity preceded agonist activation in 18% of efforts. In some intramuscular recordings, the first motor unit recruited in co-contractions was identified as that first-recruited when the same muscle was contracted voluntarily as agonist.

**Conclusions:**

Spastic co-contraction likely reflects misdirected descending supraspinal command, potentiated by stretch-related peripheral afferent signals.

**Significance**. Spastic co-contraction is an *effort-dependent*, *stretch-sensitive* phenomenon distinct from spasticity, and can severely impair force generation in spastic paresis.

## Highlights

• Spastic co-contraction reflects misdirected supraspinal command, not stretch-reflex.
• Spastic co-contraction severity is effort-dependent and can reverse joint torque.
• Muscle stretch markedly aggravates centrally driven co-contraction.

Among the various forms of muscle overactivity in spastic paresis, spasticity has been characterised as an increase in the velocity-dependent reflex response to phasic muscle stretch, recognized and measured *at rest* (Lance, 1980; Gracies, 2005). More generally, antagonist muscle overactivity is one of three cardinal disabling features in spastic paresis, alongside intrinsic muscle disorder (‘spastic myopathy’) and stretch-sensitive paresis (Gracies et al., 2025). There is an uneven distribution of these alterations around joints: paresis often predominates on one side of a given joint, while overactivity and spastic myopathy are more prominent in the antagonist (Gracies et al., 2025; Gracies et al., 2005). Spasticity, as defined above *stricto sensu*, is a primarily spinal phenomenon and has long been considered a main contributor to the movement impairment in spastic paresis (Rushworth, 1964; Mizrahi and Angel, 1979).

However, another form of muscle overactivity is pathological antagonistic co-contraction *during voluntary contraction* of the agonist (Gracies et al., 2025; Gracies et al., 2005) and has been recognized since at least the late 19th century (Nothnagel, 1872; Beaunis, 1885). Co-contraction, *i.e.*, simultaneous activity in both agonist and antagonist, is common even in normal human movement, particularly as a postural control mechanism (Levin and Dimov, 1997; Simoneau et al., 2009;Pierrot-Deseilligny and Burke, 2005) and is also observed in non-spastic ‘dystonic’ syndromes (van der Kamp et al., 1989). In spastic paresis however, many reports document that this phenomenon becomes excessive in such patients (Tardieu, 1972; Tardieu et al., 1971; McLellan, 1977; Knutsson and Richards, 1979; Knutsson and Mårtensson, 1980; Levin and Hui-Chan, 1994; Ikeda et al., 1998; Vinti et al., 2012; Vinti et al., 2013; Vinti et al., 2015; Vinti et al., 2018; Ghédira et al., 2021; Tedroff et al., 2008; Vinti et al., 2025; Canning et al., 2000). Despite this, the interpretation of its functional significance, or even its occurrence, has been disputed (Lamontagne et al., 2000; Gowland et al., 1992; Thomas et al., 1998; Newham and Hsiao, 2001; Burne et al., 2005; Lorentzen et al., 2019; Gribble et al., 2003; Enoka, 1997; Rodrigues et al., 2009; Miller and Dewald, 2012; Dewald et al., 1995; Chae et al., 2002) and its mechanisms remain the subject of conjecture (Knutsson and Richards, 1979; Knutsson and Mårtensson, 1980; Levin and Hui-Chan, 1994; Ikeda et al., 1998; Vinti et al., 2012; Vinti et al., 2013; Vinti et al., 2015; Vinti et al., 2018; Ghédira et al., 2021; Tedroff et al., 2008; Vinti et al., 2025; Canning et al., 2000; Lamontagne et al., 2000; Gowland et al., 1992; Thomas et al., 1998; Newham and Hsiao, 2001).

**Fig. 1:**
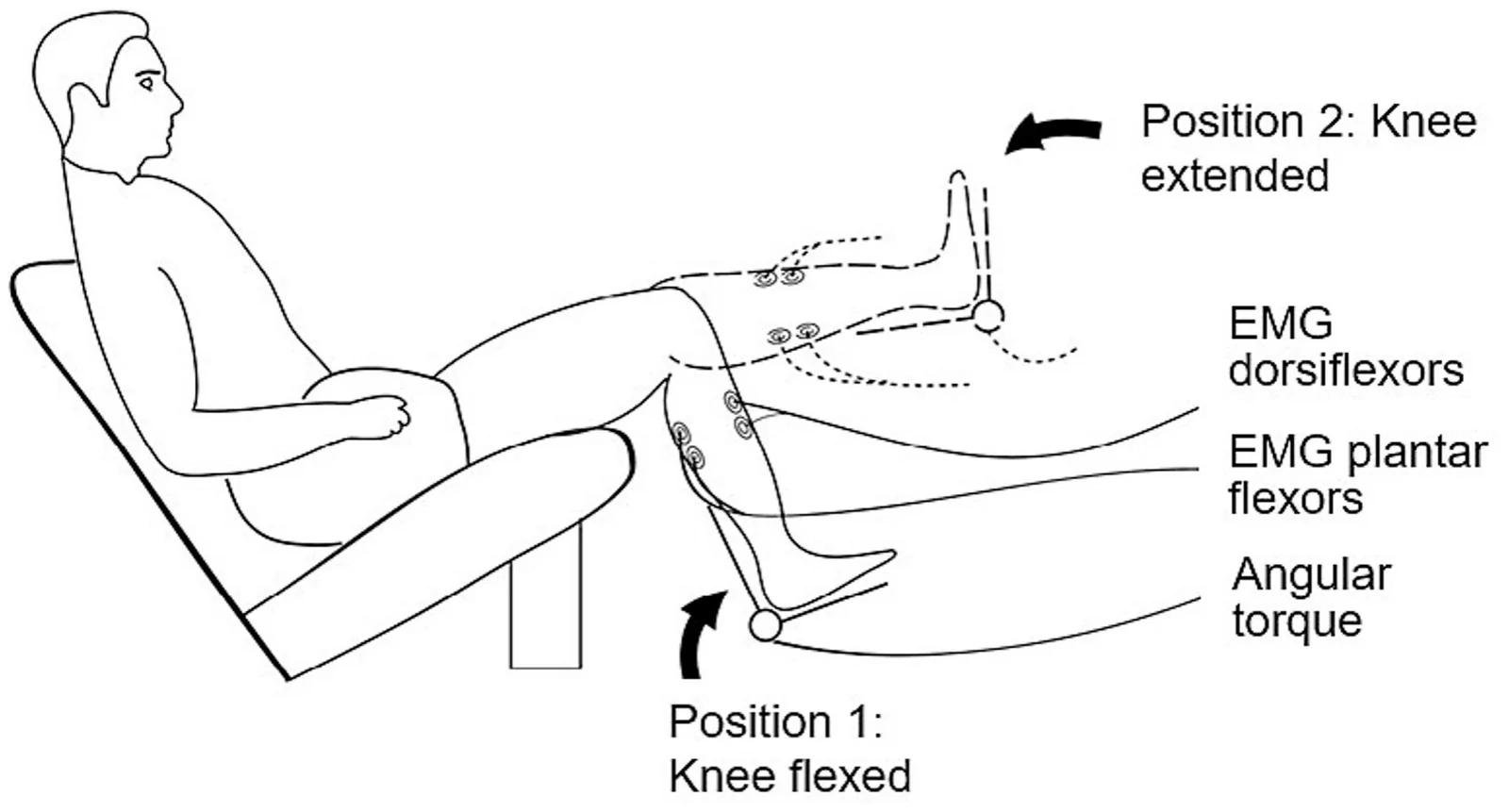
**Experimental set up** Isometric voluntary efforts in plantar and dorsiflexion were measured using an angular strain gauge with the ankle maintained at 90° of dorsiflexion in two knee positions, flexed at 90° (gastrocnemii slack) and extended (gastrocnemii stretched). Surface EMG was recorded from plantar and dorsiflexor muscles.

Here, we aimed to fill a gap in the literature (i) by demonstrating how pathological antagonistic contraction in spastic paresis can often limit or even reverse torque production, and (ii) by putting forward arguments for the underlying neurophysiological mechanisms. We used a model of *isometric* efforts at the ankle (Fig. 1), thus minimizing joint movement, an approach that is particularly suited to differentiate co-contraction from spastic responses to phasic muscle stretch. Particular aims of this study were to assess the dependence of co-contraction on changes in central commands and on changes in peripheral conditions. Specifically, by minimizing peripheral movement, the isometric paradigm made it possible to assess the effects of the intensity of voluntary command (sub-maximal *versus* maximal efforts of plantar- and dorsiflexion) and of muscle position (gastrocnemius stretched *versus* slack) on the severity of co-contraction (torque reversal *vs* no torque reversal) in plantar- and dorsiflexors in subjects with spastic paresis. We also evaluated the timing of co-contraction onset with respect to the onset of agonist activation, and added intramuscular hook-wire EMG recordings in some cases to verify the absence of cross-talk and to investigate the behaviour of single motor units.

## 1 Methods

Thirteen patients (age 55 ± 16; 1 female) with spastic paresis from stroke (hemiparesis; *n* = 12) or spinal injury (paraparesis; n = 1 who was subjected to bilateral testing so that, overall, 14 limbs were tested) participated. All patients gave informed written consent to the experimental protocols which had been approved by the Committee on Experimental Procedures Involving Human Subjects, University of New South Wales, Australia. Subjects sat (hip flexed at 80–90°) with the paretic foot strapped to a footplate connected to a force transducer (Fig. 1). The ankle was fixed at 90° dorsiflexion.

Subjects performed sub-maximal (*i.e.*, instruction ∼50% maximal) then maximal voluntary isometric plantar flexor (PF) and dorsiflexor (DF) efforts lasting ∼10 s under two conditions: knee flexed at 90° and then knee extended. In the sub-maximal efforts, subjects were requested to make an effort that they felt was 50% maximal. In total, eight subjects performed a total of 8 submaximal plantar-flexion efforts then 8 maximal efforts, plus 8 submaximal dorsiflexion efforts then 8 maximal efforts *i.e.*, a total of 32 efforts, all with knee flexed, repeated with knee extended (grand total = 64 efforts). Because the speed of contraction is typically affected in hemiparetic limbs, emphasis was put on exerting the required effort – submaximal *vs* maximal effort - rather than achieving the target level quickly. This also ensured that rapid contractions did not disturb the limb, which is important because fixation of the limb can never be absolute in human subjects, and because movement-related afferent input might cause reflex changes. The distinction between submaximal and maximal efforts was based on the effort that the subject reportedly exerted, rather than on the achieved torque output. In spastic paresis, antagonist co-contraction itself may substantially interfere with net torque generation, such that produced torque may not accurately reflect the intensity of descending motor command (Vinti et al., 2012, Vinti et al., 2015). In addition, basing effort levels on achieved force would assume a near-linear relationship between effort and force production, which is not the case even in healthy subjects. It would have required repeated maximal contractions throughout the procedures and this could have induced fatigue and modified motor recruitment patterns.

Footplate torque and PF and DF electromyograms (EMG) were monitored, the latter using pairs of surface electrodes (3 M Red Dot ECG electrodes), over PF and DF muscles at the junction upper third/lower two thirds of the leg, gain up to 50 k with a Butterworth filter 4th order, bandwidth 3.2 Hz-3.2 kHz. A ground electrode was placed over the proximal third of the medial surface of the tibia. In addition, EMG was also recorded in nine subjects using percutaneous bipolar hook-wire electrodes in tibialis anterior and gastrocnemius medialis. In these intramuscular recordings, the activity of single motor units was identified from the stability and reproducibility of motor-unit waveform morphology across repeated firings, together with a physiologically consistent discharge pattern over time. These criteria were used to distinguish local motor-unit recruitment from potential signal contamination originating from opposing muscles. In addition, the uptake area of bipolar hook-wire electrodes is ∼1–2 mm, making it unlikely that the agonist EMG was field spread from the antagonist. Data were digitized (EMG sampling frequency 1 kHz) converted and rectified using CED 1401 interface (Spike 2, CED, Cambridge, UK).

To optimize the reliability of co-contraction assessments, we used all-or-none, binary EMG and torque criteria for an ordinal classification among levels of severity, and defined 3 co-contraction levels: (1) *absent*: no detectable antagonist EMG (in surface or intramuscular wire recordings) even at high gain (up to 50 k) during agonist effort; (2) *mild*: antagonist EMG (surface or needle) during agonist effort but with torque still developing in the intended direction; (3) *severe*: antagonist EMG (surface or needle) associated with torque reversal, *i.e.*, unintended antagonist activity overwhelmed the intended agonist contraction (*e.g.*, PF torque during a DF effort). These all-or-none criteria were intentionally chosen to minimize interpretative ambiguity.

Any shifts from one category to another with changes in joint position (two positions, knee flexed or extended) or with effort level, maximal *vs* sub-maximal were assessed by one experienced investigator (JMG) familiar with the recording system and the analysis procedure, based on retrospective visual inspection only. Reliance on all-or-none criteria (presence/absence of antagonist EMG and torque reversal) and the one assessor were adopted to reduce interpretative variability.

### 1.1 Statistics

All statistical tests were non-parametric, and paired since they compared the same subjects/limbs under two conditions of effort (maximal and sub-maximal), or under two conditions of gastrocnemius stretch at maximal effort. The two questions to be answered by non-parametric statistics in this study were:

1. Is antagonistic co-activation dependent upon the amount of agonist effort?

2. Is antagonistic co-activation dependent upon the amount of stretch imposed on the co-contracting antagonist?

The latter question could be answered by our protocol only for plantar flexor co-activation during dorsiflexor efforts, since the gastrocnemius muscles were the only muscle group undergoing two different amounts of stretch between the knee flexed and the knee extended positions, the ankle remaining fixed at 90°.

Three complementary statistical approaches were used to address these two questions and assess the robustness of findings:

1. Considering the three-level ordinal variable defining co-contraction severity as (1) No co-contraction; (2) mild co-contraction (antagonist EMG without torque reversal); and (3) Severe co-contraction (torque reversal), we used Wilcoxon signed rank tests (Wilcoxon, 1946) to determine whether co-contraction severity differed between maximal *vs* sub-maximal efforts and between knee flexed *vs* knee extended. Effect sizes were calculated for significant results and interpreted as small (*r* = 0.10 to <0.30), moderate (*r* = 0.30 to <0.50), or large (*r* ≥ 0.50) (Fritz et al., 2012; Tomczak and Tomczak, 2014).
2. Two dichotomous variables of co-contraction severity were also considered: (1) No co-contraction *versus* Co-contraction; and (2) No or mild co-contraction *versus* Severe co-contraction. The effects of maximal *vs* sub-maximal effort and gastrocnemius stretch *vs* no gastrocnemius stretch were then evaluated using McNemar testing (McNemar, 1947) or two-tailed binomial testing depending on the condition. (Cohen, 1988; Zar, 2010)
3. Finally, *two-tailed binomial tests* were performed to explore whether *changes* in co-contraction severity were equally distributed between increased and decreased severity of co-contraction, or differed from an expected balance of 50% changes in either direction. Effect sizes were calculated for significant findings (see Supplementary Statistical Note).

In addition, for descriptive purposes we compared sub-maximal and maximal efforts for peak rectified antagonist EMG and peak torque using binary classifications: increase *vs* no increase in antagonist EMG, and decline *vs* no decline of torque in the desired direction when effort was increased (see small characters in Table 2). Decline was defined from the pattern seen in the torque recordings, as: (1) a decrease in the intended torque; or (2) torque reversal; or (3) increase in unintended (reverse) torque. Further technical details concerning test selection and effect-size calculations are provided in the Supplementary Statistical Note. Statistical tests used SPSS 26.0 (Chicago, IL). Statistical significance was at *p* = 0.05.

## 2 Results

Subject/limb clinical characteristics and EMG recording techniques are displayed in Table 1. Subjects 11 and 12 were studied two months after the lesion (*i.e.*, in the subacute phase), and the other subjects were studied in the chronic phase, *i.e.*, over six months after the lesion. They all presented with a moderate to severe degree of plantar flexor spasticity, as indicated by the Tardieu Scale data (Table 1). Qualitatively the responses seen in each limb of the paraparetic patient were similar to those in the hemiparetic patients, and the results are pooled below.

**Table 1:** Patient details - recording techniques.

Subject/limb #	Gender	Age (yrs)	Time since lesion (mo)	Lesion Type	Lesion site	Ankle Foot Orthosis	Plantar flexor Spasticity (Tardieu)	Plantar flexor Tone (Ashworth)	EMG Recording technique
1	M	57	17	I	R brainstem	0	95°,3	3	S; HW GM + TA
2	M	61	16	I	L MCA	+	95°,3	2	S
3	M	59	7	I	L MCA	+	100°,3	2	S
4	M	26	24	I	R internal capsule	0	110°,3	2	S; HW GM
5	M	60	42	H	L temporoparietal	0	100°,3	3	S
6	M	72	6	I	L MCA	+	105°,2	2	S; HW GM + TA
7	M	37	72	I	Spinal Cord (L)	0	80°,3	3	S; HW GM
8	M	37	72	I	Spinal Cord (R)	0	75°,3	3	S
9	M	60	6	I	L internal capsule	0	90°,3	2	S; HW GM + TA
10	M	67	84	I	L MCA	+	85°,2	2	S
11	M	55	2	I	R internal capsule	+	90°,3	3	S; HW GM + TA
12	M	70	2	H	R brainstem	+	110°,4	3	S; HW GM + TA
13	M	63	36	I	R internal capsule	0	100°,3	3	S; HW TA
14	F	23	12	H	R temporoparietal	0	90°,3	2	S; HW GM

M, male; F, female; MCA, Middle Cerebral Artery; S, surface; HW, hook wire; GM, gastrocnemius medialis; TA, tibialis anterior.

The results are displayed in Table 2, Table 3, and a summary of the main inferential statistical comparisons, including the number of observations, direction of effect, *p*-value, and effect size where applicable, is provided in Table 4.

**Table 2:** Influence of effort on co-contraction in spastic paresis.

	Knee Flexed								Knee Extended							
	*PFxr effort*				*DFxr effort*				*PFxr effort*				*DFxr effort*			
			*Deterioration*				*Deterioration*				*Deterioration*				*Deterioration*	
*Subject/limb #*	*Sub max*	*Max*	*EMG*	*Torque*	*Sub max*	*Max*	*EMG*	*Torque*	*Sub max*	*Max*	*EMG*	*Torque*	*Sub max*	*Max*	*EMG*	*Torque*
1	**++**	**++**	+	+	**0**	**0**	0	0	+	+	+	0	**++**	**++**	+	+
2	**+**	**++**	+	+	**+**	**+**	+	0	0	+	+	+	**++**	**++**	+	+
3	**+**	**+**	+	0	**+**	**+**	+	0	0	+	+	0	**+**	**++**	+	+
4	**+**				**+**	**+**	+	0	+	+	+	0	**++**	**++**	+	+
5	**+**	**+**	+	0	**+**	**+**	+	0	+	+	+	0	**+**	**+**	+	0
6	**+**	**++**	+	+	**0**	**+**	+	0	0	+	+	0	**++**	**++**	+	+
7	**0**	**+**	+	0	**+**	**+**	+	0	+	+	+	0	**0**	**+**	+	0
8	**+**	**+**	+	0	**+**	**+**	+	0	+	+	+	0	**0**	**+**	+	0
**Cocontraction**	7/8	7/7			6/8	7/8			5/8	8/8			6/8	8/8		
**Torque reversal**	1/8	3/8			0/8	0/8			0/8	0/8			4/8	5/8		
**Deterioration EMG**			7/7				7/8				8/8				8/8	
**Deterioration torque**				3/7				0/8				1/8				5/8

**0**, absence of co-contraction; **+**, mild co-contraction; **++**, severe co-contraction; Deterioration EMG, increase in antagonist EMG; Deterioration torque, decrease in agonist torque, torque reversal or increase in unintended (reverse) torque. The left and right legs of the paraparetic subject are numbered 7 and 8.

**Table 3:** Influence of stretch on co-contraction in spastic paresis (maximal isometric efforts).

	Dorsiflexor efforts		Plantar flexor efforts	
	*Plantarflexor CC*		*Dorsiflexor CC*	
Subject #	Knee flexed	Knee extended	Knee extended	Knee flexed
1	0	++	+	++
2	+	++	+	++
3	+	++	+	+
4	+	++	+	+
5	+	+	+	+
6	+	++	+	++
7	+	+	+	+
8	+	+	+	+
9	+	++	0	+
10	+	++	+	+
11	+	++	+	+
12	+	+	+	+
13	+	+	+	+
14	+	++	+	+
**Severe CC**	**0/14**	**9/14**	**0/14**	**3/14**
	KE *vs* KF p = 0.004 (two-tailed Wilcoxon, confirmed by binomial test)		KE *vs* KF p = 0.046 (Wilcoxon)	

CC = co-contraction; 0, absence of co-contraction; +, mild co-contraction (no torque reversal); ++, severe co-contraction (with torque reversal).

**Table 4:** Summary of statistical analyses of co-contraction severity.

Question	Variable	Comparison and outcome	Unit of analysis	n	Direction and descriptive result	Statistical test	p-value	Effect size
**Effect of effort intensity**	**Ordinal 3-grade variable of co-contraction severity**	Max *vs* submax; All knee positions and effort directions	Paired efforts	31	Max effort: 1.23 ± 0.50 *vs* 0.90 ± 0.65 at submax effort	Wilcoxon	0.002	*r* = 0.57, large
		Max *vs* submax; Knee flexed, both effort directions	Paired efforts	15	Max effort: 1.13 ± 0.52 *vs* 0.87 ± 0.52 at submax effort	Wilcoxon	0.046	*r* = 0.52, large
		Max *vs* submax; Knee extended, both effort directions	Paired efforts	16	Max effort: 1.31 ± 0.48 *vs* 0.94 ± 0.77 at submax effort	Wilcoxon	0.014	*r* = 0.61, large
		Max *vs* submax; Plantar flexor CC severity during dorsiflexion efforts, both knee positions	Paired efforts	16	Max effort: 1.25 ± 0.58 *vs* 1.00 ± 0.73 at submax effort	Wilcoxon	0.046	r = 0.52, large
		Max *vs* submax; Dorsiflexor CC severity during plantar flexion efforts, both knee positions	Paired efforts	15	Max effort: 1.20 ± 0.41 *vs* 0.80 ± 0.56 at submax effort	Wilcoxon	0.014	r = 0.61, large
	**1st dichotomous variable**	Max *vs* submax; Absence *vs* presence of any CC (mild or severe)	Paired efforts	31	Prevalence of CC increased from 74% (23/31) at submax effort to 97% (30/31) at max effort	Wilcoxon; McNemar	0.008; 0.016	*r* = 0.48, moderate (Wilcoxon)
	**2nd dichotomous variable**	Max *vs* sub max; Absence or mild CC *vs* severe CC	Paired efforts	31	Prevalence of severe CC increased from 16% (5/31) at submax effort to 26% (8/31) at max effort	Wilcoxon; McNemar	0.08; 0.25	NS
	**Direction of changes in co-contration severity**	Changes in ordinal CC severity from submax to max effort	Paired efforts	31	10 severity increases *vs* 0 decrease	Binomial	0.002	Not measurable (10/0)
**Effect of knee position / gastrocnemius stretch on plantar flexor co-contraction during max dorsiflexion efforts**	**Ordinal 3-grade variable of co-contraction severity**	Knee extended *vs* knee flexed	Limbs	14	Knee extended: 1.64 ± 0.50 *vs* 0.93 ± 0.27 knee flexed	Wilcoxon	0.004	*r* = 0.86, very large
	**2nd dichotomous variable**	Knee extended *vs* knee flexed: Severe plantar flexor CC during max dorsiflexion efforts	Limbs	14	Prevalence of severe CC increased from 0% (0/14) with knee flexed to 64% (9/14) with knee extended	Wilcoxon; McNemar	0.003; 0.004	r = 0.86, very large (Wilcoxon)
	**Direction of changes in co-contration severity**	Changes in plantar flexor CC severity from knee flexed to knee extended	Limbs	14	9 severity increases *vs* 0 decrease	Binomial	0.004	Not measurable (9/0)
**Effect of knee position on dorsiflexor co-contraction during max plantar flexion efforts**	**Ordinal three-grade variable of co-contraction severity**	Knee extended *vs* knee flexed	Limbs	14	Knee flexed: 1.21 ± 0.43 *vs* 0.93 ± 0.27 knee extended	Wilcoxon	0.046	*r* = 0.53, large
	**2nd dichotomous variable**	Knee extended *vs* knee flexed	Limbs	14	Prevalence of severe CC decreased from 21% (3/14) with knee flexed *vs* 0% (0/14) with knee extended	Wilcoxon; McNemar	0.08; 0.25	NS
	**Direction of changes in co-contration severity**	Changes in dorsiflexor co-contraction severity from knee extended to knee flexed	Limbs	14	4 severity increases *vs* 0 decrease	Binomial	0.12	Not measurable (4/0)

CC = co-contraction; max, maximal efforts; submax, submaximal efforts.

Using two graded effort levels (Table 2) and two different knee positions (Table 3), antagonist contraction (*i.e.*, at least *mild* cocontraction) was seen in surface EMG recordings in at least one of the tested effort levels or knee positions in each of the 14 limbs tested during both plantar or dorsiflexion efforts. Antagonist co-contraction was confirmed using intramuscular wire recordings in all 9 subjects in whom this was examined.

The sample sizes for the statistical tests depended on the comparison performed. For comparisons between knee positions, n refers to the number of limbs contributing paired observations across the knee-flexed and knee-extended positions (n = 14). For comparisons between effort intensity levels, n refers to the number of paired efforts available for comparison between submaximal and maximal conditions (n = 31), in which each paired effort corresponded to a given limb, effort direction, and knee position.

### 2.1 Effect of effort level

Table 2 indicates the level of co-contraction (absent, mild or severe) in the two positions knee flexed and knee extended, compared for sub-maximal and maximal efforts.

When using the Wilcoxon signed rank test on the ordinal variable 0, *no co-contraction*, 1 *mild co-contraction* and 2 *severe co-contraction*, across both knee positions and effort directions (n = 31 efforts), co-contraction severity was greater during maximal than sub-maximal efforts (*p* = 0.002; Wilcoxon, n = 31), with severity scores of 1.23 ± 0.50 (mean ± SD, n = 31) and 0.90 ± 0.65 (n = 31) respectively. The effect size, calculated as r = Z/√N, was 3.16/5.57 = 0.57 (*i.e.*, a large effect). This effect was confirmed whichever the knee position and whichever the effort direction:

When considering the position Knee Flexed only, across both effort directions, co-contraction severity also increased from submaximal efforts (0.87 ± 0.52; *n* = 15) to maximal efforts (1.13 ± 0.52, n = 15; *p* = 0.046, Z = 2.00; n = 15). The effect size, r = Z/√N, was 2.00/3.87 = 0.52 (*i.e.*, a large effect). Similarly, in the position Knee Extended only, across both effort directions, co-contraction severity increased from submaximal efforts (0.94 ± 0.77; *n* = 16) to maximal efforts (1.31 ± 0.48, *n* = 16; *p* = 0.014, Z = 2.45; n = 16). The effect size, r = Z/√N, was 2.45/4 = 0.61 (*i.e.*, a large effect).

When distinguishing effort directions, during dorsiflexor efforts across both knee positions co-contraction severity increased from submaximal efforts (1.00 ± 0.73; n = 16) to maximal efforts (1.25 ± 0.58, n = 16; *p* = 0.046, Z = 2.00; n = 16). The effect size, r = Z/√N, was 2.00/3.87 = 0.52 (*i.e.*, a large effect). During plantar flexor efforts across both knee positions co-contraction severity also increased from submaximal efforts (0.80 ± 0.56; *n* = 15) to maximal efforts (1.20 ± 0.41, n = 15; *p* = 0.014, Z = 2.45; n = 15). The effect size, r = Z/√N, was 2.45/4 = 0.61 (*i.e.*, a large effect).

Using the first dichotomous variable – absence *vs* presence of co-contraction (mild or severe) - the prevalence of *any* co-contraction (mild or severe), across both muscle groups and knee positions, increased from 74% (23 of 31 efforts) during submaximal efforts to 97% (30 of 31) during maximal efforts, (*n* = 31 paired efforts; *p* = 0.008, Z = 2.65, Wilcoxon; *p* = 0.016, McNemar, Table 2)*.* The effect size of the Wilcoxon result was r = Z/√N, *i.e.* 2.65/5.57 = 0.48 (*i.e.*, a moderate effect).

Using the second dichotomous variable – absence or mild co-contraction *vs severe* co-contraction - the prevalence of severe co-contraction across both muscle groups and knee positions (n = 31 paired efforts) was 16% (5 of 31 efforts) during submaximal efforts and 26% (8 of 31 efforts) during maximal efforts, not significantly different (*p* = 0.08, Z = 1.73, Wilcoxon; *p* = 0.25, McNemar*; n* *=* 31 paired efforts).

**Fig. 2:**
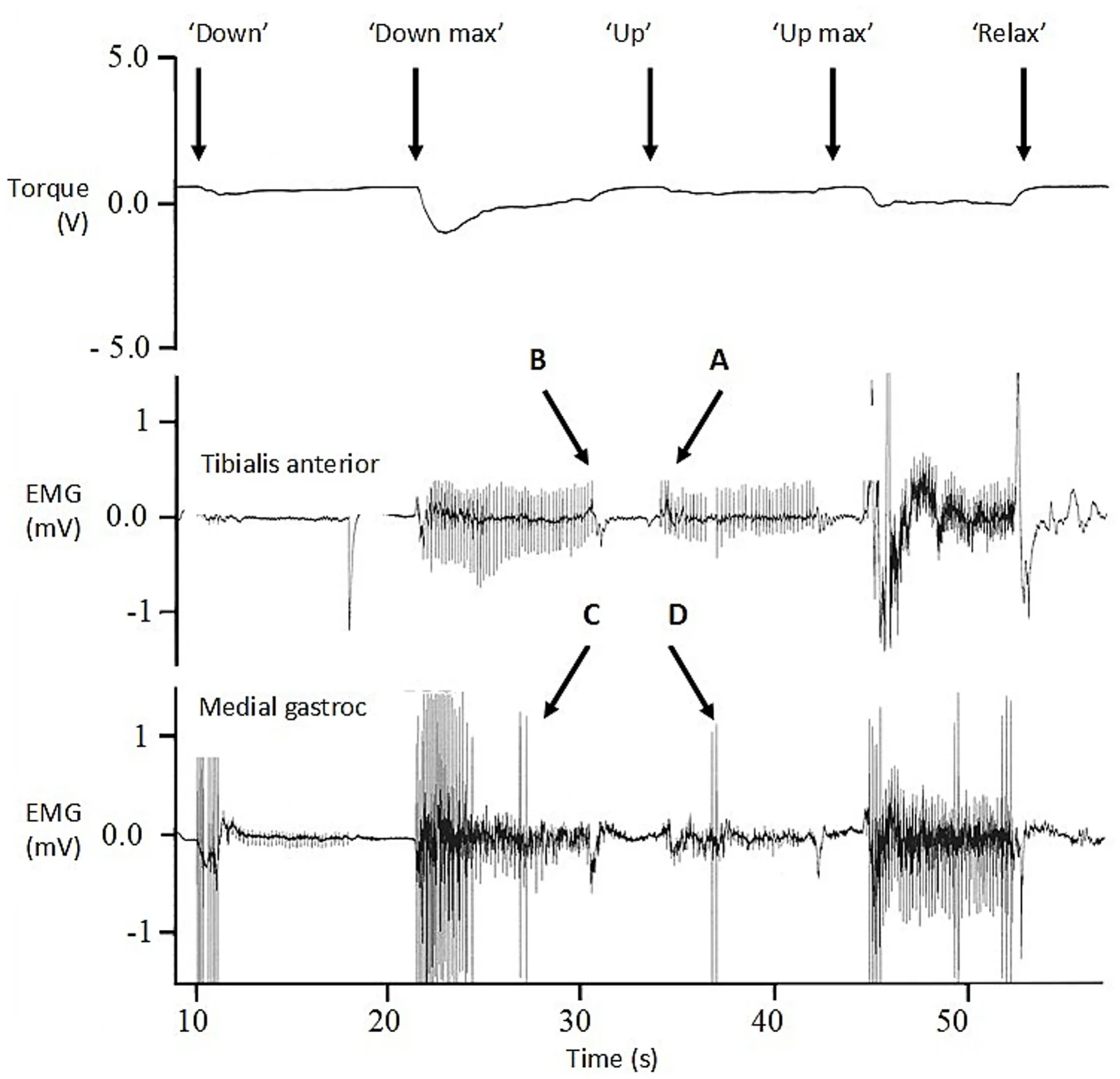
**Increase in Spastic Co-contraction with effort - data from one subject, knee extended.** Upper trace, angle ankle torque (downward = plantar flexor torque); Intramuscular wire recordings from tibialis anterior (middle trace) and medial gastrocnemius (lower trace). Dorsiflexor co-contraction was observed during maximal plantar flexor effort (“Down Max”) but not during non-maximal effort (“Down”). Plantar flexor co-contraction was observed in both non-maximal (“Up”) and maximal (“Up Max”) efforts but considerably worse in the latter. A, B, C, D, individual motor units on an expanded trace in Figure 3.

When examining the direction of changes in co-contraction severity from submaximal to maximal effort across both muscle groups and knee positions (*n* = 31 efforts), co-contraction severity increased from *absent* to *mild* or from *mild* to *severe* in 10 out of 31 cases (32%) overall, whereas there was no case (0%) of a decrease in severity. All 10 changes were therefore towards a worsening of the co-contraction and none towards improvement. A two-tailed binomial test with *p* = 0.5 and *n* = 10 showed that twice the probability of observing this distribution was 0.002. The effect size, theoretically given by the ratio 10/0, could not be estimated because of the zero denominator. Descriptive results (small print in Table 2) confirmed that the greater the effort, the more severe the co-contraction: with greater efforts, peak antagonist EMG amplitudes increased in 30 out of 31 cases (97%) and agonist ankle torque *decreased* in 9 of 31 cases (29%), with torque reversal in 3 of 31 cases (10%; Table 2, small characters). Fig. 2, obtained using intramuscular wire recordings from tibialis anterior and medial gastrocnemius in Subject 6, illustrates the increase in co-contraction with effort.

### 2.2 Characteristics of antagonist recruitment

#### 2.2.1 Recruitment pattern

**Fig. 3:**
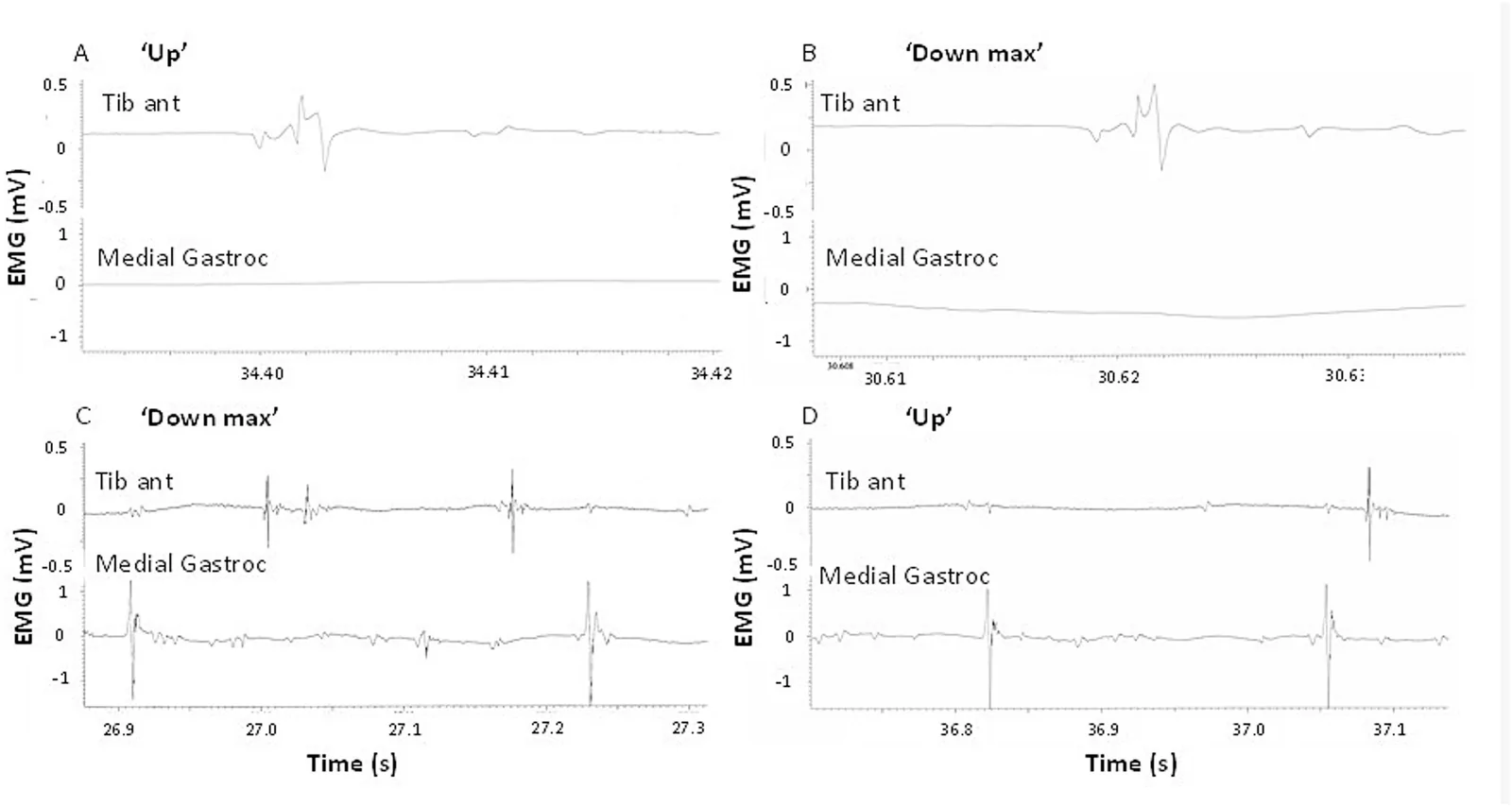
**Recruitment pattern during agonist effort and antagonist co-contraction.** Expansions of EMG traces from Figure 2. The first motor unit recruited in tibialis anterior when activated as an agonist during non-maximal dorsiflexor effort (3A – “Up”) is also the first unit recruited in the unwanted co-contraction of this muscle when acting as an antagonist during maximal plantar flexor effort (3B – “Down Max”). Conversely, a large motor unit recruited in medial gastrocnemius during a maximal plantar flexor effort (3C – “Down Max”), is rapidly recruited in its co-contraction during non-maximal dorsiflexor effort (3D – “Up”).

Fig. 3 shows data from Fig. 2, with the EMG traces expanded to show the morphology of the motor units recruited when the muscle was first activated as an agonist and subsequently as a co-activated antagonist. The tibialis anterior motor unit recruited first when this muscle was acting as an agonist during a sub-maximal dorsiflexor effort (Fig. 3A - “Up”) was also recruited first in the unwanted co-activation during maximal plantar flexor effort (Fig. 3B - “Down Max”). Consistently, in gastrocnemius medialis a large unit recruited in maximal plantar-flexor effort was also rapidly recruited during its co-activation in a sub-maximal dorsiflexor effort.

While this study was not intended to analyse single motor unit behaviour systematically, a similar recruitment pattern between descending agonist command and antagonist co-activation for a given muscle was detected in other recordings made using intramuscular wire electrodes, much as in this Figure.

#### 2.2.2 Recruitment timing

**Fig. 4:**
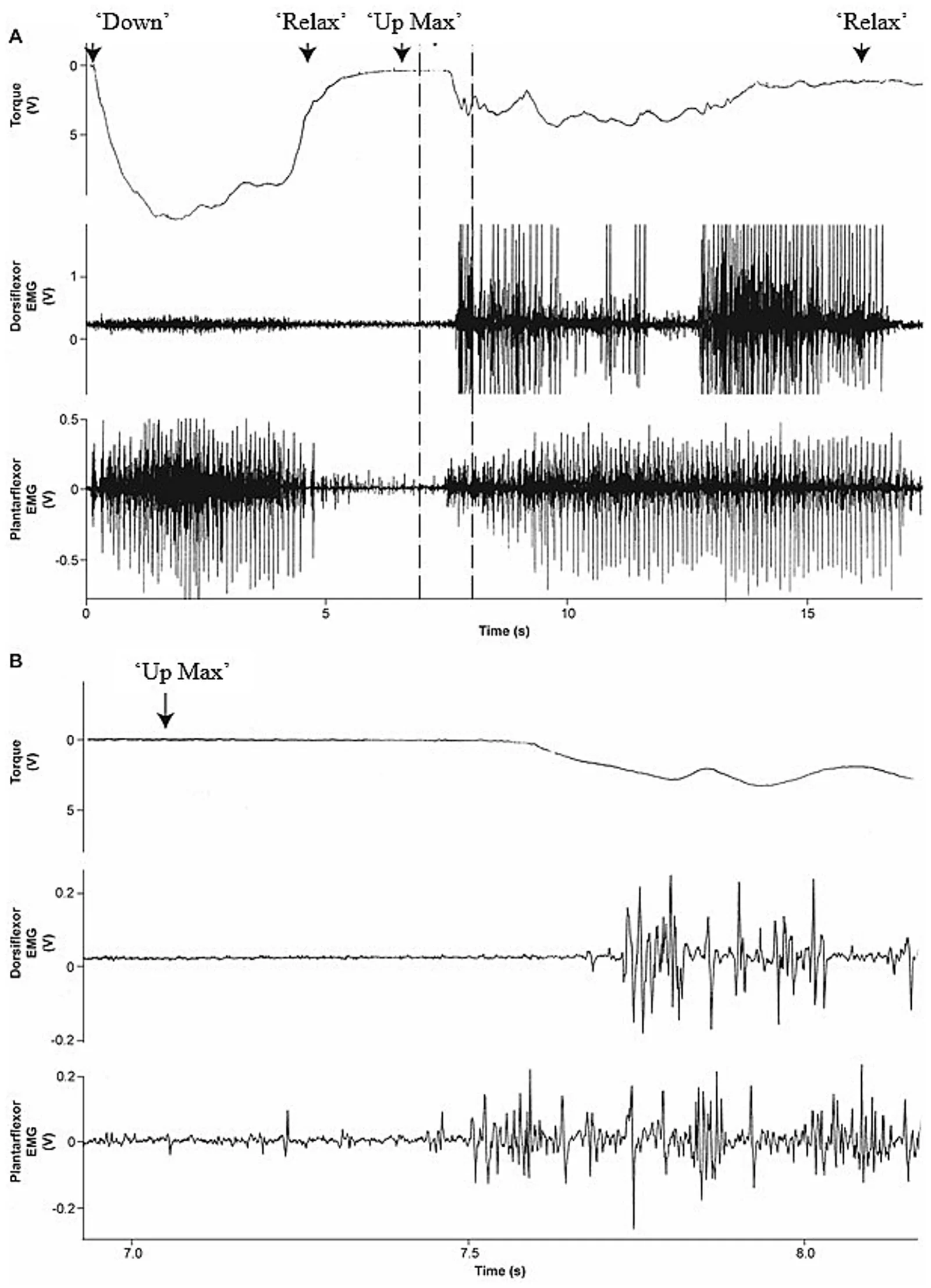
**Timing of antagonist co-contraction (individual data).** Set up as above. In the lower set, EMG and torque traces were expanded around the onset of dorsiflexor recruitment during a dorsiflexor effort. Expanded traces show that the antagonist plantar flexor recruitment begins about 200 ms earlier than any agonist dorsiflexor unit.

Fig. 4 illustrates severe plantar-flexor co-contraction during dorsiflexion efforts. Panel 4B shows the EMG and torque traces expanded around the onset of dorsiflexor recruitment, with EMG in the antagonist plantar flexors beginning about 200 ms before agonist dorsiflexor recruitment. Such antagonist EMG activity beginning *prior* to the intended agonist contraction occurred in 18% of recordings (10 out of 56).

## 3 Stretch-sensitivity

Table 3 grades each maximal effort tested according to the level of co-contraction (absent, mild or severe) in the two positions knee flexed and knee extended for each effort direction.

### 3.1 Plantar flexor co-contraction during dorsiflexor efforts

Using the three-level ordinal classification of co-contraction severity variable – *0, no cocontraction; 1, mild cocontraction and 2, severe cocontraction* - plantar flexor co-contraction during maximal dorsiflexor efforts was markedly more severe with the *knee extended* than with the *knee flexed.* Mean severity scores were 1.64 ± 0.50 with the knee extended *versus* 0.93 ± 0.27 with the knee flexed (*n* = 14; *p* = 0.004; Z = 2.89; Wilcoxon, *n* = 14). The effect size, calculated as r = Z/√N, was 2.89/3.74 = 0.86 (*i.e.*, a very large effect).

When considering the first dichotomous variable – absence *vs* presence of any co-contraction, whether mild or severe - the prevalence of any plantar flexor co-contraction during maximal dorsiflexor efforts was 93% (13 of the 14 efforts) with the knee flexed *vs* 100% (14 of 14 efforts) with the knee extended (NS).

Using the second dichotomous variable however – absence or mild co-contraction *vs* severe co-contraction - the prevalence of *severe* plantar flexor co-contraction during maximal dorsiflexor efforts was 0% (0 of 14 efforts) with the knee flexed *versus* 64% (9 of 14 efforts) with the knee extended (*n* = 14; *p* = 0.003, Z = 3.0, Wilcoxon; p = 0.004, McNemar/ exact binomial test, n = 14). The effect size, provided by the formula r = Z/√N, was 3.0/3.74 = 0.86 (*i.e.*, a very large effect).

Co-contraction severity increased with knee extension in 9 out of 14 limbs (64%), and there was no case of a decrease in severity (0%). Among the nine limbs showing a change in co-contraction severity, all nine changes were therefore towards worsening of the co-contraction and none towards improvement (*n* = 9 discordant limbs). A two-tailed binomial test with *p* = 0.5 and n = 9 showed that twice the probability of observing this distribution was *p* *=* 0.004. The effect size, theoretically given by the ratio 9/0, could not be estimated because of the zero denominator.

### 3.2 Dorsiflexor co-contraction during plantar flexor efforts

Using the three-level ordinal severity variable *0, no co-contraction, 1 mild co-contraction and 2 severe co-contraction*, dorsiflexor co-contraction during maximal plantar flexor efforts was significantly more severe with the *knee flexed* than with the *knee extended.* Mean severity scores were 1.21 ± 0.43 with the knee flexed *versus* 0.93 ± 0.27 with the knee extended (*n* = 14 limbs; *p* = 0.046; Z = 2.00, Wilcoxon). The effect size, provided by the formula r = Z/√N, was 2.00/3.74 = 0.53 (*i.e.*, a large effect, but one that was not confirmed by the complementary tests).

Using the first dichotomous variable – absence *vs* presence of any co-contraction (mild or severe) - the prevalence of *any* dorsiflexor co-contraction (mild or severe) during maximal plantar flexor efforts was 100% in the knee flexed position (14 of the 14 efforts tested) *vs* 93% in the knee extended position (not significant).

Using the second dichotomous variable – absence or mild co-contraction *vs* severe co-contraction - the prevalence of *severe* dorsiflexor co-contraction during maximal plantar flexor efforts was 21% (3 of 14 efforts) with knee flexed *versus* 0% with knee extended (0 of n = 14 limbs; *p* = 0.08, Z = 1.73, n = 14, Wilcoxon; *p* = 0.25, McNemar /binomial distribution).

When limbs went from the *knee extended* to the *knee flexed*, the severity of co-contraction increased with knee flexion in 4 out of 14 limbs (29%), whereas no decrease in severity (0%) was observed. Among the 4 limbs showing a change in co-contraction severity, all 4 changes were therefore towards increased severity with knee flexion and none towards decreased severity (*n* = 4 discordant limbs). A two-tailed binomial test with *p* = 0.5 and *n* = 0 + 4 = 4 showed that twice the probability of getting 0 or fewer changes from *Mild Co-contraction* to *No co-contraction* or from *Severe Co-contraction* to *Mild or No Co-contraction* out of 4 changes was 0.12 (NS).

## 4 Discussion

The present study of voluntary isometric ankle dorsiflexor and plantar flexor efforts in spastic paresis shows a high prevalence and negative functional impact of antagonist co-contraction, particularly during maximal efforts when co-contraction almost always occurred. The change in the severity of plantar-flexor co-contraction with knee position was remarkable, capable of reversing the intended torque into plantar flexion in 64% of dorsiflexor efforts when the knee was extended *vs* 0% of dorsiflexor efforts with knee flexed. When effort was increased from sub-maximal to maximal, the intended contraction torque decreased in 29% of tests and was even reversed in 10%.

### 4.1 Physiological co-contraction

In healthy subjects, physiological co-contraction of antagonists is often observed as a postural anticipatory mechanism or for learning a skill and improving accuracy (Levin and Dimov, 1997; Pierrot-Deseilligny and Burke, 2005; Gribble et al., 2003). Such skill-learning associated co-contraction usually decreases with practice (Gribble et al., 2003; Enoka, 1997). However physiological co-contraction may increase during acute fatigue when it is probably due to a breakdown of motor control, producing a mistimed and misdirected motor command, much as probably occurred in the paretic subjects studied here (Rodrigues et al., 2009). A number of features may distinguish the antagonist co-contraction described in paretic patients in this study, from fatigue-enhanced physiological co-contraction. These include the absence of fatigue-inducing efforts prior to the present experiments and the ability of the unintended antagonist co-contraction in spastic paresis to completely overwhelm the willed contraction of the agonist.

### 4.2 Antagonist co-contraction in spastic paresis

It is well known that corticospinal lesions due to stroke result in impaired selection of the appropriate muscle synergies for a willed movement, with activation of groups of muscles not normally involved in the movement (Miller and Dewald, 2012; Dewald et al., 1995). In particular, overt co-contractions of *antagonists* have been reported in many studies of paretic upper or lower limbs (Tardieu, 1972; McLellan, 1977; Vinti et al., 2012; Vinti et al., 2025; Dewald et al., 1995; Chae et al., 2002). The isometric paradigm used here was intended to rule out the possibility that the unwanted antagonist contraction resulted from an uninhibited reflex response to stretch (see Methods).

### 4.3 Misdirection of the supraspinal drive

Three observations support the hypothesis that these antagonistic co-contractions are primarily linked to altered descending motor commands rather than being solely generated by peripheral reflex mechanisms. First, the onset of co-activated antagonist recruitment occasionally preceded agonist recruitment. Under isometric conditions with the agonist not yet activated, there should be no increase in peripheral afferent feedback that could reflexly trigger recruitment of the antagonist (either stretch, or an afferent volley due to agonist recruitment). Secondly, the first motor unit recruited during voluntary agonist recruitment was also often recruited first when the muscle was activated in a co-contraction. This would be expected if the same command pathways were responsible for the pathological antagonistic co-contraction and the agonist contraction, *i.e.*, a descending pathway of supraspinal origin. Admittedly this might also occur if reflex inputs recruited motoneurones in the same sequence as voluntary drives in spastic subjects, but again the isometric situation limited potential reflex-eliciting afferent inputs (Ashworth et al., 1967). Finally, co-contraction consistently increased with the intensity of effort, despite unchanged joint position and peripheral conditions, supporting a direct relationship between descending motor drive and antagonist recruitment. Taken together, these observations provide indirect physiological evidence supporting the view that antagonistic co-contractions in spastic paresis reflect a misdirected descending supraspinal drive rather than reflex contractions triggered peripherally. This represents our preferred interpretation of the present findings, though we concede that we have not directly demonstrated a misdirected supraspinal command. The possibility that fusimotor neurones might have been activated prior to the appearance of EMG and thereby provided an afferent input in advance of EMG is not consistent with data from healthy subjects (Burke, 1981; Pierrot-Deseilligny and Burke, 2005) or from patients with spasticity (Hagbarth et al., 1973; Wilson et al., 1999; Macefield, 2013).

### 4.4 Peripheral modulation of co-contraction by stretch

Although the present findings support a primarily supraspinal origin of spastic co-contraction, they also suggest that its severity may be secondarily accentuated by peripheral stretch-related mechanisms. In particular, tonic stretch imposed on the gastrocnemius muscles (knee extended position) aggravated its co-contraction during dorsiflexion efforts. Joint receptor activation could also contribute to this phenomenon, though most human joint afferents appear to act mainly as stress receptors activated near the extremes of movement range (Burke et al., 1988). This marked stretch-dependent aggravation of co-contraction is one of the features distinguishing the present form of spastic co-contraction from the physiological co-contraction observed during normal movement, postural stabilization or motor learning (Levin and Dimov, 1997; Pierrot-Deseilligny and Burke, 2005; Gribble et al., 2003; Aagaard et al., 2000; Smith, 1981).

Furthermore, it is remarkable that antagonist co-contraction was of much greater functional impact and magnitude in the plantar flexors during dorsiflexion efforts than in the opposite situation. Of the two antagonist muscles groups, the plantar flexors are particularly prone to spastic myopathy and contracture in spastic paresis (Ramsay et al., 2014; Pradines et al., 2022). This raises the possibility that antagonist co-contraction may predominate in the more severely affected, *i.e.* structurally more altered muscle, of an agonist-antagonist couple (Pradines et al., 2022).

**Fig. 5:**
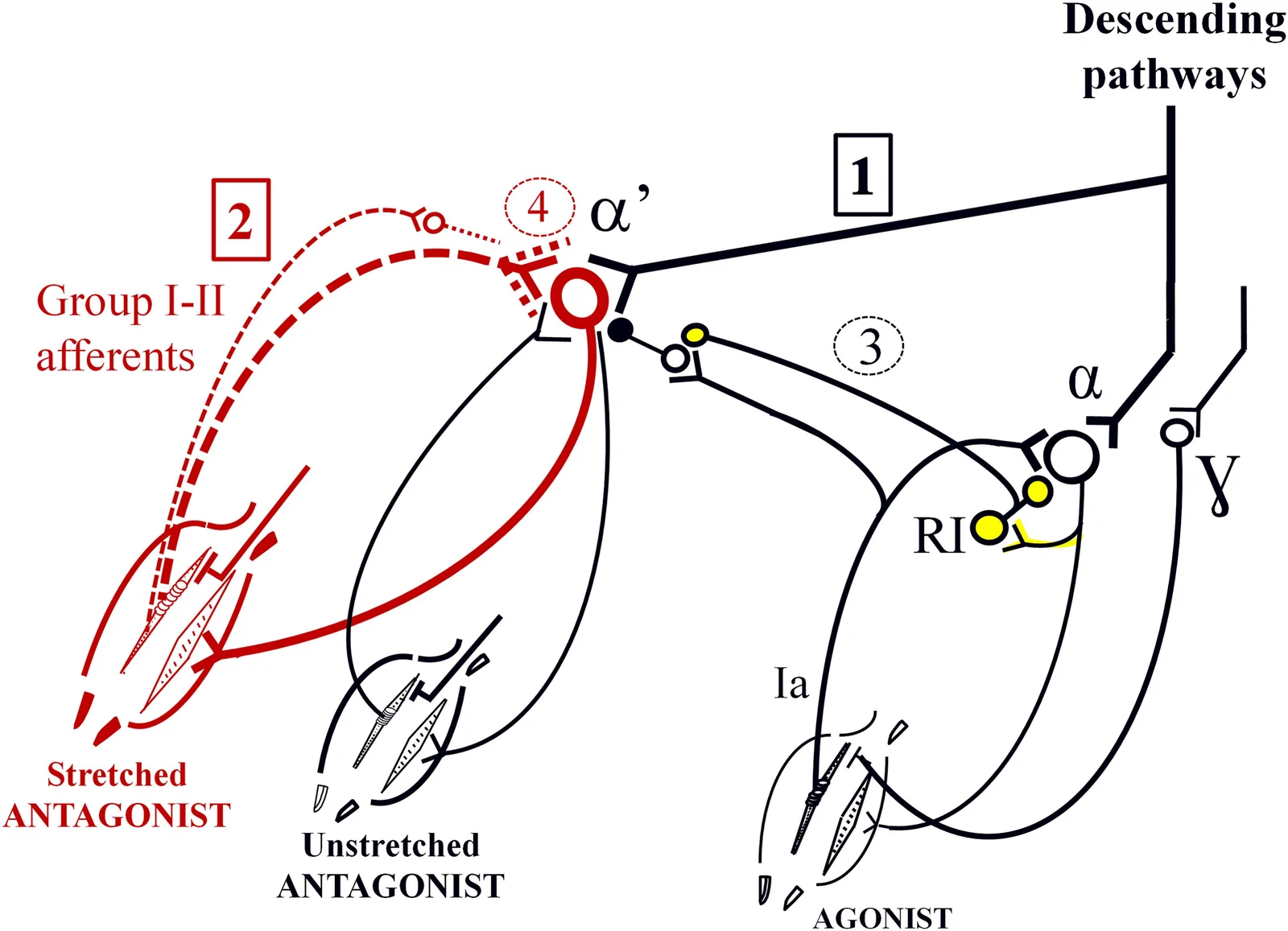
**Fig 5. Schematic (conceptual diagram) summarizing the potential mechanisms causing and worsening Spastic Co-contraction.** Inverted arrows represent excitatory synapses; full circles represent inhibitory synapses; RI, Renshaw inhibition. Proposed mechanisms are numbered from 1 to 4. The two mechanisms supported by the present findings are numbered in bold, while additional putative mechanisms, not evaluated in the present experiments, are circled in dashed lines. 1. Misdirection of the supraspinal descending drive; 2. Aggravation by stretched position of the co-contracting antagonist, recruiting Group I and Group II excitatory afferents; 3. Failure of reciprocal inhibition due to increased Renshaw inhibition from the agonist motoneurone; 4 Synaptic sensitization occurring due to chronic, repeated stimulation of primary and secondary spindle afferents from a contractured antagonist. A misdirected supraspinal drive will be more likely to bring antagonist motoneurones to firing threshold if there is reduced reciprocal Ia inhibition, even more so with firing from Group I-II afferents from a stretched antagonist muscle. For simplicity, the Group I and II projections to common propriospinal neurones excitatory to homonymous motoneurones are not specifically illustrated.

One likely mechanism underlying this stretch-sensitivity involves increased activation of muscle spindle afferents in muscles that have been chronically shortened and stiffened through prolonged hypo-mobilization in an unstretched position, as is typically the case for gastrocnemius in hemiparesis (Gioux and Petit, 1993; Rosant et al., 2006). In spastic paresis, reduced muscle extensibility as part of spastic myopathy may increase baseline spindle discharge for a given amount of muscle lengthening when the muscle is placed under tonic stretch (Gioux and Petit, 1993; Rosant et al., 2006). This increased spindle afferent activity is probably due to enhanced mechanical transmission of stretching forces through a stiffer muscle than to increased fusimotor drive, for which no convincing evidence has been reported in spastic muscles (Hagbarth et al., 1973; Wilson et al., 1999; Macefield, 2013). Enhanced gastrocnemius group I and II afferent input might then facilitate the inappropriate recruitment of gastrocnemius motoneurones during voluntary effort directed to tibialis anterior (Fig. 5) (Marque et al., 2005). Over time, repeated activation of these stretch-related afferent pathways could contribute to activity-dependent synaptic potentiation within the spindle-afferent to motor neuron pathways, potentially lowering the threshold for gastrocnemius motoneurone recruitment and further amplifying co-contraction severity (Kandel and Tauc, 1964).

### 4.5 Potential aggravation of co-contraction by failure of reciprocal inhibition

In addition to altered descending motor command and stretch-related peripheral amplification, an intrinsically deregulated spinal mechanism could come into play to worsen co-contractions. The abnormal co-contraction in spastic paresis may involve dysfunctional recurrent (Renshaw) inhibition from the contracting agonist, which may be increased at rest in patients with spastic paresis (Smith, 1981; Katz and Pierrot-Deseilligny, 1982; Little and Halar, 1985; Shefner et al., 1992; Taylor et al., 1984). As paretic patients also lose the ability to modulate recurrent inhibition during voluntary effort (Katz and Pierrot-Deseilligny, 1982; Little and Halar, 1985; Shefner et al., 1992; Taylor et al., 1984), a greater background level might play a role in spastic co-contraction, because recurrent collaterals from Renshaw cells suppress Ia inhibitory interneurones directed to the antagonist (Hultborn et al., 1971a; Hultborn et al., 1971b; Hultborn et al., 1971c). Further studies have indeed demonstrated a lack of the normal increases in reciprocal Ia inhibition and presynaptic inhibition on Ia afferents directed to the antagonist during voluntary contractions in spastic paresis (Morita et al., 2001). These abnormalities would facilitate the expression of any descending drives that were misdirected to the antagonist (Fig. 5). By comparison, in healthy subjects involuntary co-contractions are associated with *increased* presynaptic inhibition of Ia afferents and increased fusimotor drive (Llewellyn et al., 1990). However, in most cases of spastic paresis of both spinal and cerebral origin, presynaptic inhibition on Ia afferents is decreased at rest (Gracies et al., 2025; Pierrot-Deseilligny and Burke, 2005), and there is no evidence for increased fusimotor drive (Hagbarth et al., 1973; Wilson et al., 1999; Macefield, 2013). Thus, altered recurrent and reciprocal inhibitory mechanisms could contribute to the abnormal expression of spastic co-contraction, through mechanisms distinct from those underlying physiological co-contraction.

### 4.6 Importance of the more contractured muscle around a joint as a conditioner of the transmission of descending command?

Regarding dorsiflexor co-contractions during plantar flexor efforts, the first of the series of statistical tests did indicate greater severity of dorsiflexor co-contractions in the knee flexed position, *i.e.*, when gastrocnemius were not under stretch. If this is reproduced in other studies, it would suggest a way to protect against dorsiflexor co-contraction, *i.e.*, by placing the agonist gastrocnemius muscles under stretch (Pradines et al., 2019; Ramsay et al., 2014). This observation may have less of a functional impact than the difficulty picking up the foot during the swing phase. But greater efficiency in plantar flexing when the knee is extended would constitute positive functionality. This mechanism could have practical importance, but will need to be verified by further research.

Indeed, if protection against co-contraction of the less contractured muscle is confirmed in further studies, there might be a role for the more contractured muscle in modulating the expression of descending motor command: in other words a role as a *conditioner* of the transmission of descending command. Imposing stretch on that muscle would aggravate its co-contraction when acting as an antagonist to the intended command, while this would protect against co-contraction of the opposing muscle when acting as an agonist. The specific contribution of spastic myopathy to this modulation remains to be directly investigated.

### 4.7 Functional significance and therapeutic avenues

There have been suggestions that antagonist co-contraction may serve a compensatory role in some contexts in hemiparesis, such as during the stance phase of gait, where co-contraction might be seen as an adaptive strategy contributing to joint stabilization and postural control (Lamontagne et al., 2000; Chow et al., 2012; Souissi et al., 2018). The functional impact of co-contractions necessarily depends on the motor task. During phases requiring active movement in an open kinetic chain, such as ankle dorsiflexion during the swing phase of gait, inappropriate activation of the plantar flexors would oppose the intended movement and interfere with foot clearance and limb advancement. In this context, excessive co-contraction would be functionally detrimental, and it has been correlated with the sense of effort and with weakness around the joint (Vinti et al., 2013; Vinti et al., 2015).

In real life, a number of “near-isometric” situations may resemble the isometric paradigm tested in this study, requiring active dorsiflexion when the knee is almost fully extended and the ankle dorsiflexed, *e.g.*, during normal stance when leaning backwards, or during the mid-stance of gait. Plantar flexor co-contractions could then delay or impede toe-off in the subsequent pre-swing and swing phases of gait. Other examples include before bending the hip and lifting the foot when advancing up a flight of steps, or the leg posture of a driver who wishes to switch the foot from the one pedal to another. Unwanted co-contraction has been found at other joints (Tardieu, 1972; McLellan, 1977; Vinti et al., 2012; Vinti et al., 2025), and could thus be an important contributor to functional disability in a range of voluntary movements in spastic paresis. Indeed, we argue that it would create more disability than spasticity *per se.* (Rushworth, 1964; Mizrahi and Angel, 1979; Ghédira et al., 2022).

Classical teaching has been that movements in spastic paresis are impaired by *spasticity*, *i.e.*, are impeded by the reflex contraction of an antagonist muscle to the phasic stretch produced by movement (Rushworth, 1964; Mizrahi and Angel, 1979; Ashworth, 1964), and this has led to a search for chemical or physical therapies focused on dampening stretch reflexes (Bütefisch et al., 2000; Bobath and Bobath, 1950; Gracies et al., 2002). Some of these therapies have included synaptic depressors, which are also known today as inhibitors of brain plasticity (Bütefisch et al., 2000; Gracies et al., 2002). A primary goal of reducing co-contraction would, however, lead to different therapeutic strategies than if the goal was merely to reduce stretch reflex activity.

First, the primary need to reshape the descending command may redirect the focus of the neurorehabilitation community on retraining *unassisted* voluntary movement. A good example is the use of rapid or maximal amplitude alternating movement exercises, which may gradually refocus the motoneuronal command and reduce antagonist co-contraction in spastic paresis, as has been suggested in early robotic therapy trials (Bütefisch et al., 1995; Hu et al., 2007). A second potential therapeutic strategy to diminish co-contraction in spastic paresis may involve daily forceful stretch of the more contractured of the two antagonists, with the goal of re-lengthening muscle, thus rendering its spindles less sensitive to stretch and thereby reduce their ability to enhance co-contraction (Pradines et al., 2019; Pradines et al., 2024). Finally, botulinum toxin injections into the more contractured of two co-contracting antagonists could reduce co-contraction even in non-injected muscle on the opposite side of the joint, despite the absence of toxin diffusion between sides. This has been shown with reduction of triceps brachii co-contractions after injections of biceps brachii (Gracies et al., 2009; Vinti et al., 2012; Delcamp et al., 2022), enhancing active elbow extension capacities (Gracies et al., 2015). In the lower limb at the ankle, clinical experience is also available (Boulay et al., 2023), with demonstrated enhancement of reciprocal inhibition after botulinum toxin injection (Aymard et al., 2013). One hypothesis is that the toxin-induced decrease in recurrent inhibition on the injected muscle (Hagenah et al., 1977; Hagenah, 1979; Hamjian and Walker, 1994; Tyler, 1963) might indeed release reciprocal Ia inhibition from the injected agonist to the antagonist during agonist effort (Hultborn et al., 1971a), thus explaining decreased co-contraction in the non-injected antagonist as well.

### 4.8 Spastic co-contraction as part of the spectrum of muscle overactivity in spastic paresis

Besides spastic co-contraction during voluntary effort, patients with spastic paresis are recognizable *at rest* by two key neurological features, the first of which is measurable at bedside: *spasticity*, *i.e.* enhanced velocity-dependent responses to phasic stretch, and *spastic dystonia*, which is chronic tonic contraction, detected in the absence of phasic stretch and in the absence of voluntary effort (Gracies et al., 2025; Lorentzen et al., 2018). Of the two phenomena, only spastic dystonia is clinically significant, as it can be psychologically and physically disabling due to the body deformities it causes.

*During voluntary effort*, these patients develop *spastic co-contraction*, with the fundamental features elucidated in the present study: potential onset before agonist recruitment in isometric situation and worsening when the co-contracting antagonist is under stretch. The present findings support the hypothesis that spastic co-contraction primarily reflects a misdirection of the supraspinal descending drive, worsened by stretch receptor stimulation in the co-contracting muscle. Functionally, this type of overactivity is most disabling, correlated with the sense of effort and with weakness around the joint (Vinti et al., 2013; Vinti et al., 2015). Additional types of motor neuronal overactivity of spastic paresis comprise spasms at rest and synkinesis during effort (Gracies et al., 2025).

### 4.9 Study limitations

Several methodological limitations should be acknowledged. First, effort was defined according to how the subjects perceived their voluntary motor commands rather than by achieved torque. This was for good reasons outlined in the Methods. The experimental protocol was designed to characterize co-contraction in maximal *vs* submaximal efforts rather than the trial-to-trial reproducibility of submaximal torque, and no formal measure of within-subject consistency of submaximal efforts was obtained. Therefore, some uncertainty may remain regarding the consistency of voluntary efforts across experimental conditions. Secondly, although co-contraction severity was classified using deliberately simple, all-or-none criteria based on antagonist EMG activity and torque reversal, all recordings were evaluated by the same experienced examiner, and no formal assessment of intra-rater reliability was performed. Future studies should formally evaluate the reproducibility of this classification over time. Thirdly, we did not directly demonstrate the supraspinal origin of the misdirected descending command, but inferred that from the observed recruitment patterns. Future studies combining the present paradigm with assessments of corticospinal excitability on the antagonist pathway, spinal inhibitory reflexes, and peripheral afferent contributions could help disentangle the respective roles of descending, spinal and peripheral mechanisms in the initiation and modulation of antagonist co-contraction.

## 5 Conclusion

The high prevalence and functional impact of the inappropriate antagonist recruitment demonstrated here need to be better recognized in spastic paresis. This phenomenon is distinct from spasticity *per se*, and its functional consequences in paretic patients may not respond to treatments primarily aimed at the reflex hyperexcitability of spasticity. Given its sensitivity to stretch of the relevant muscle, this phenomenon should be termed ‘spastic co-contraction’.

## Funding

For this study, JMG was supported by a Fellowship from the 10.13039/501100001677Institut National de la Santé et de la Recherche Médicale (France) and the 10.13039/501100000925National Health and Medical Research Council (Australia).

## Declaration of competing interest

The authors declare that they have no conflict of interest.
