# Supplementary material for: Spastic co-contraction: A misdirected central command
Source: Clin Neurophysiol Pract. 2026 Aug 15;11:714–26. doi: 10.1016/j.cnp.2026.08.005 (PMC13520632; doi:10.1016/j.cnp.2026.08.005)
Supplement: Supplementary file 1 [file mmc1.docx]

***Supplementary statistical note***

All statistical tests were non-parametric, and paired since they compared the same subjects/limbs under two conditions of effort (maximal and sub-maximal), or under two conditions of gastrocnemius stretch at maximal effort. The two questions to be answered by non-parametric statistics in this study were:

1. Is antagonistic co-activation dependent upon the amount of agonist effort?

2. Is antagonistic co-activation dependent upon the amount of stretch imposed on the co-contracting antagonist?

Regarding the latter question, our protocol could only answer it for plantar flexor co-activation during dorsiflexor efforts, since the gastrocnemius muscles were the only muscle group undergoing two different amounts of stretch between the knee flexed and the knee extended positions (the ankle remaining fixed at 90°).

Three different statistical methods addressed each of these two questions and verified robustness of results:

1.	Considering the three-level ordinal variable defined as 1. No co-contraction; 2 mild co-contraction (antagonist EMG without torque reversal); 3. Severe co-contraction (torque reversal), we used Wilcoxon signed ranked tests (Wilcoxon, 1946) to determine whether this ordinal variable behaved differently in the two conditions maximal *vs* sub-maximal effort on one hand, and in the two conditions knee flexed *vs* knee extended on the other hand. Effect sizes of any significant results were provided by the formula r = Z statistic divided by square root of the sample size (N) (Fritz et al, 2012; Tomczak and Tomczak, 2014). The recommended interpretation values for r, are: 0.10 - < 0.3 (small effect), 0.30 - < 0.5 (moderate effect) and >= 0.5 (large effect; Fritz et al, 2012; Tomczak and Tomczak, 2014).

2.	Considering two dichotomous variables 1. No co-contraction; 2. Co-contraction (either mild or severe); and 1. No or mild co-contraction; 2. Severe co-contraction; effects of the categorical situations defined above (maximal *vs* sub-maximal effort and gastrocnemius stretch *vs* no gastrocnemius stretch), were evaluated using McNemar testing (McNemar,1947) or two-tailed binomial testing depending on the condition. (Cohen, 1988; Zar, 2010; Fritz et al, 2012)

3.	Finally, a series of *exact two-tailed binomial tests* were performed to explore whether the changes in co-contraction severity were equally distributed between increased and decreased severity of co-contraction, or differed from an expected balance of 50% changes in either direction. If significance of the two-tailed binomial test results was observed, effect sizes were estimated as ES = ψ = max(A/B, B/A) (A, observed proportion of changes in one direction, e.g., severity increase/B, observed proportion of changes in the other direction, e.g., severity decrease; Cohen, 1988; Zar, 2010).

In addition, for descriptive purposes we compared sub-maximal and maximal efforts for peak rectified antagonist EMG and peak torque using binary classification: increase *vs* no increase in antagonist EMG, and decline *vs* no decline of torque in the desired direction when effort was increased (see small characters in Table 2). Decline was defined from the pattern seen in the torque recordings, as: (1) a decrease in the intended torque; or (2) torque reversal; or (3) increase in unintended (reverse) torque. Statistical tests used SPSS 26.0 (Chicago, IL). Statistical significance was at p=0.05.
